# Differences between urban and rural hedges in England revealed by a citizen science project

**DOI:** 10.1186/s12898-016-0064-1

**Published:** 2016-07-22

**Authors:** Laura Gosling, Tim H. Sparks, Yoseph Araya, Martin Harvey, Janice Ansine

**Affiliations:** 1 Imperial College London, South Kensington, London, SW7 2AZ UK; 2 Coventry University, Priory Street, Coventry, CV1 5FB UK; 3 The Open University, Milton Keynes, Buckinghamshire, MK7 6AA UK; 4 Birkbeck, University of London, London, WC1E 7HX UK

**Keywords:** Hedges, Invertebrates, Roadsides, Species richness, Volunteers, Woody species, Citizen science

## Abstract

**Background:**

Hedges are both ecologically and culturally important and are a distinctive feature of the British landscape. However the overall length of hedges across Great Britain is decreasing. Current challenges in studying hedges relate to the dominance of research on rural, as opposed to urban, hedges, and their variability and geographical breadth. To help address these challenges and to educate the public on the importance of hedge habitats for wildlife, in 2010 the Open Air Laboratories (OPAL) programme coordinated a hedge-focused citizen science survey.

**Results:**

Results from 2891 surveys were analysed. Woody plant species differed significantly between urban and rural areas. Beech, Holly, Ivy, Laurel, Privet and Yew were more commonly recorded in urban hedges whereas Blackthorn, Bramble, Dog Rose, Elder and Hawthorn were recorded more often in rural hedges. Urban and rural differences were shown for some groups of invertebrates. Ants, earwigs and shieldbugs were recorded more frequently in urban hedges whereas blowflies, caterpillars, harvestmen, other beetles, spiders and weevils were recorded more frequently in rural hedges. Spiders were the most frequently recorded invertebrate across all surveys. The presence of hard surfaces adjacent to the hedge was influential on hedge structure, number and diversity of plant species, amount of food available for wildlife and invertebrate number and diversity. In urban hedges with one adjacent hard surface, the food available for wildlife was significantly reduced and in rural hedges, one adjacent hard surface affected the diversity of invertebrates.

**Conclusions:**

This research highlights that urban hedges may be important habitats for wildlife and that hard surfaces may have an impact on both the number and diversity of plant species and the number and diversity of invertebrates. This study demonstrates that citizen science programmes that focus on hedge surveillance can work and have the added benefit of educating the public on the importance of hedgerow habitats.

## Background

Hedges are familiar structures in the British landscape. They are a boundary or linear feature of shrubs and/or trees that is subject to some degree of management [1, 2] and are a distinct part of British cultural heritage [3].

The archetypal British landscape of a patchwork of small fields surrounded by hedges may be a feature of the period that follows the Great Enclosure (1750–1850) when 200,000 miles of hedges were planted [4]. However, there is clear evidence that the overall amount of hedges in Great Britain has decreased over the past 70 years [5–7]. Modernisation of agriculture after World War II led to considerable hedge removal and changes to management practices. Recognition of this led to a spate of research into the role of hedges in the countryside and the publication of a book specifically on hedges in the *new naturalist* series [6]. Another significant loss of hedges occurred in the latter part of the 20th century [8]. The 1990 Countryside Survey [9] reported that the length of hedges in Britain had decreased by 23 % between 1984 and 1990. This alarming discovery led to hedges being designated as a priority habitat for conservation in the 1994 UK Biodiversity Action Plan (now superseded by the UK post-2010 Biodiversity Framework [10]). However, despite no discernible change in hedge length between 1990 and 1998 [11], the 2007 Countryside Survey reported a further decrease in hedges, estimating a 1.7 % reduction in the total length of woody linear features in Great Britain since 1998 [5]. A reduction of 6.2 % in the same time frame was recorded for managed hedges, representing a loss of 31,000 km of hedge. Reasons for this are largely related to changes in cultural functions [2] and the over- and under-management of hedges [5, 12].

Hedges are largely man-made, often as a result of boundary delineation, agricultural practices such as stock control, or for provision of resources [2, 13]. They are aesthetically pleasing landscape features in both rural and urban areas and offer soft functions such as colours, smells and patterns [3]. Particularly in urban areas they may also provide privacy [3], function as noise barriers [14] and, broadly, vegetation in cities may help to mitigate air pollution [15].

Hedges are important ecologically and are highly valued for their ability to provide food and shelter for a wide range of vertebrates and invertebrates [2, 16–18]. Their structure and composition has an important influence on wildlife presence and abundance. Modelled relationships between the different structural components of a hedge and the animals that use them have shown that each component (e.g. trees, shrub layer) has value for different animal species throughout the year [19]. Other studies have focussed on particular species. For example, trees in hedges provide habitats for bats [20] and large moth species [21], and gaps in hedge structure influences bank vole abundance [22] and beetle populations [23]. The plants that make up a hedge also affects animal diversity with both herbivorous and detritivorous invertebrates and their associated predators and parasites being affected by floral composition [24]. They are also considered to be ‘corridors’ between areas such as woodlands, although it is noted that their function in this respect is lacking in empirical evidence [25–27]. Furthermore, hedges provide significant ecosystem services. They provide a regulating service through controlling water flow and preventing soil erosion [2, 28]. They provide a supporting service through soil nutrient retention [28] and provide habitats for pollinating insects, essential for arable farming [29]. They also provide an important cultural service through their heritage value as part of the British landscape. Hedge biodiversity is influenced *inter alia* by the management regime used: cutting frequency and timing can affect the diversity of hedge flora [30] and fauna [31, 32] and the food (flowers and berries) available for wildlife [12], while land use adjacent to the hedge also affects the diversity of both flora and fauna [28].

One of the significant challenges for current research on hedges is that there is considerable geographical breadth and variability in hedges. Furthermore, many of the studies highlighted thus far refer to hedges in rural areas, and in particular to farmland hedges. However, very few studies focus on urban hedges, although studies on urban ecology and biodiversity may cover hedges implicitly. The countryside survey, one of the most in-depth studies of hedges in the UK [8], does not cover urban areas and therefore the biodiversity value of urban hedges is not well known. Faiers and Bailey [33] examined canalside hedges and noted that urban hedges scored poorly for biodiversity and structure compared with rural hedges along the same 20 km stretch of canal. Other studies which have looked at the ecological differences between urban and rural environments noted the importance of landscape features and green space in providing valuable habitats in increasingly urbanised landscapes (e.g. [34, 35]) but did not necessarily look at hedge habitats.

To gain geographical coverage in environmental research, scientists are increasingly using citizen science as a tool to gather data [36–38]. This is demonstrated by a large array of research in which members of the public and non-experts collect data on a range of topics and submit these data for further interrogation [36]. This method has the core benefits of enabling scientists to collect data from areas they cannot normally access and on a large geographical scale [39], although issues of data quality should be addressed [36, 40]. Nevertheless, citizen science can be used to provide a broad overview of phenomena and, furthermore, it can be used to engage people in science and environmental monitoring, creating a legacy for future conservation.

This paper looks at the results from a citizen science hedge survey, coordinated by the Open Air Laboratories (OPAL) programme and specifically, compares urban and rural hedges.

## Methods

OPAL began in December 2007 with a grant from the UK Big Lottery Fund. OPAL aims to meet dual ambitions of encouraging more people to explore their local natural environment while also providing useful data which can be used for research [41].

With OPAL, participants of all ages and abilities carry out surveys on a range of environmental topics. The surveys have clear instructions and are designed to be self-explanatory [42] and simple to complete. In September 2010, OPAL launched the OPAL Biodiversity Survey, which asked participants to examine hedges and the biodiversity found in them. Although there are many examples of hedge surveys being organised at a local level, often using the methodologies outlined in the Hedgerow Survey Handbook [1] this OPAL survey was the first England-wide citizen science survey to address the wildlife value of hedges.

The survey was undertaken through four activities: an activity to describe the hedge’s features and components; an activity to note down how much food (berries, nuts) was present in the hedge; an activity to note any evidence of animals living there; and an activity to determine what invertebrates were found in the hedge.

Surveys were organised and often overseen by locally recruited staff (Community Scientists, see [42] and biodiversity mentors, see [43]), group leaders and school teachers. Participants in the surveys included volunteers affiliated to a community or voluntary organisation, youth groups, school groups, and groups of families and friends. Survey participants could also download a survey pack from the OPAL website [44] and take part in the survey independently of organised groups. Participants were provided with survey packs and were guided to find suitable local hedges to monitor. The survey pack included a field guide, a recording booklet and information on how to identify common hedge plants and invertebrates to varying taxonomic levels. Taxa included in the survey were selected on the basis that they were likely to be encountered and were reasonably distinctive for easy identification by untrained surveyors. For additional species identification support, participants were also guided to use the iSpot website [45], an OPAL website developed by The Open University to help people develop their interest in wildlife.

Survey participants were asked to select a three-metre stretch of hedge that was “typical of the whole hedge” [46] and to record the information listed in Table 1. The majority of surveys were entered online by the participants; a small number were entered by OPAL staff. Some responses were entered in the form of free text and these required a certain amount of editing for consistency and spelling. Participants identified the location of their hedge by pinpointing on an online map and these locations were recorded in the database in the form of latitude and longitude. ArcMap [47] and its ‘clip’ tool were used to extract surveys conducted in England only. Then the ‘selection’ tool was used to identify sites as urban or non-urban (rural) according to the 2001 census by the Office of National Statistics. The number of woody species, the number of invertebrate groups, and the total number of recorded individual invertebrates were also determined.

**Table 1:** Summarised recorded variables in the survey

Question category	Answer variables
Type of recording group	School, volunteer group, family or friends
Weather	Sunny, cloudy but no rain, raining
Location	
Surrounding area	Urban, garden, park, school, farmland, grassland, wood or forest, other
Land use on both sides of the hedge	Crops, grassland, hard surface (unspecified), garden, woodland, waterway, cannot see (other side)
Structure of hedge	Bushes, bushes and trees, trees
Gaps in hedge	None, a few, more gaps than hedge
Hedge shape	Untrimmed, leggy, laid, neatly trimmed, heavily trimmed
Features in the hedge	Fence, ditch, bank, undisturbed strip, wall
Hedge height	In four categories from <1 m to >3 m
Hedge width	In three categories from <1 m to >2 m
Hedge length	In four categories from <5 m to >50 m
Hedge plant species	Presence/absence of 12 woody species, (see Table 3)
Numbers of berries, nuts or flowers	In four categories from <10 to >1000
Invertebrates	Counts of invertebrates in 24 named groups (see Table 4)
Size of any holes in the ground	In five categories from <2 cm to >30 cm
Other wildlife seen	Free text

### Hedge scoring system

As part of the survey, a scoring system was created that would enable an assessment to be made of the potential that each hedge had for supporting a range of biodiversity (i.e. a quality score), and to provide meaningful feedback to survey participants. A number of systems have previously been developed to generate numerical scores for particular habitats. These may be based on the measured traits of particular species (e.g. [48]) or may assign values to particular habitat characteristics in order to compare different sites and assess their quality or condition (e.g. [49]). There have also been studies on how to classify hedges (e.g. [50, 51]) but only a few attempted to score hedges, such as Hedgelink’s (a partnership of 19 government, conservation and countryside organisations) management decision score [52], or to develop criteria for determining hedge importance ([53] cited in [54]). The OPAL Biodiversity Survey allowed for three scores to be created for: hedge structure/shape; provision of food for wildlife (plant species present, potential for flowers and fruits); and animal diversity based on the species found by the surveyors. Details on how scores were derived are listed in Table 2. This method has some parallels with the condition assessment approach, exemplified for grasslands by Robertson and Jefferson [49]. The method was tested using hypothetical data and then refined with data from field trials which confirmed that higher scores were generated for the better “quality” hedges. Since the three test scores were not highly positively correlated, and since each score may independently indicate a feature of benefit to wildlife, the three separate scores were retained, rather than combined into a single amalgamated score.

**Table 2:** Derivation of hedge structure score, food for wildlife score and animal diversity score

Question category	Answer variables	Score
Hedge structure score—sum of the following seven elements		
1. Structure of hedge	Line of bushes	4
	Line of trees	3
	Bushes and trees	5
2. Gaps in hedge	No gaps	5
	A few gaps	3
	More gaps than hedge	1
3. Shape of hedge: average of all that were recorded	Neatly trimmed	2
	Untrimmed	4
	Heavily cut	1
	Leggy	1
	Laid or coppiced	5
4. Other features: sum of all features recorded	Wall	1
	Fence	0
	Ditch	1
	Bank	1
	Undisturbed strip	2
5. Height of hedge	<1 m	1
	1–2 m	3
	2–3 m	5
	>3 m	4
6. Width of hedge	<1 m	1
	1–2 m	3
	>2 m	5
7. Length of hedge	<5 m	2
	5–20 m	3
	20–50 m	4
	>50 m	5
Food for wildlife score—sum of the following two elements		
1. Hedge food species: sum of all recorded species. Sum multiplied by 2 if hedge shape recorded as “untrimmed”, or by 1.5 if shape recorded as “neatly trimmed” (providing “untrimmed” was not recorded)	Beech	1
	Bramble	5
	Blackthorn	5
	Dog Rose	5
	Elder	3
	Hawthorn	5
	Hazel	2
	Holly	4
	Ivy	4
	Laurel	1
	Privet	2
	Yew	2
2. Numbers of flowers/berries	<10	1
	10–100	4
	100–1000	7
	>1000	10
Animal diversity score—sum of the following two elements		
1. Numbers of different types (see Table 4) of invertebrate: sum scores for all invertebrate types recorded.	Recorded as present	2
	1–2	2
	3–5	2.5
	6–10	3
	11–50	3.5
	51–500	4
	>500	5
2. Presence of holes	<2 cm	5
	2–5 cm	10
	5–10 cm	15
	10–30 cm	20
	>30 cm	25

### Statistical analysis

Differences between urban and rural hedges in: (i) the proportions containing specific woody species and invertebrate groups were compared using Chi squared contingency tables, and (ii) the mean number of woody species, mean number of invertebrates, mean hedge structure score, mean wildlife food score and mean animal diversity score by independent samples t tests. A comparison between the number of invertebrate groups and the number of woody hedge species was made using Spearman rank correlation.

Hedge structure score, wildlife food score, animal diversity score, numbers of invertebrate groups and numbers of woody hedge species were further examined using analysis of variance (ANOVA). For each of the five variables an ANOVA was undertaken examining differences in urban and rural hedges, hard surface types adjacent to the hedge, and the interaction between urban/rural and hard surfaces. Hard surfaces were categorised as 0, 1 or 2 depending on how many sides of the hedge were hard surfaces. All analyses and graphs were generated in SPSS version 22 [55].

## Results

**Fig. 1:**
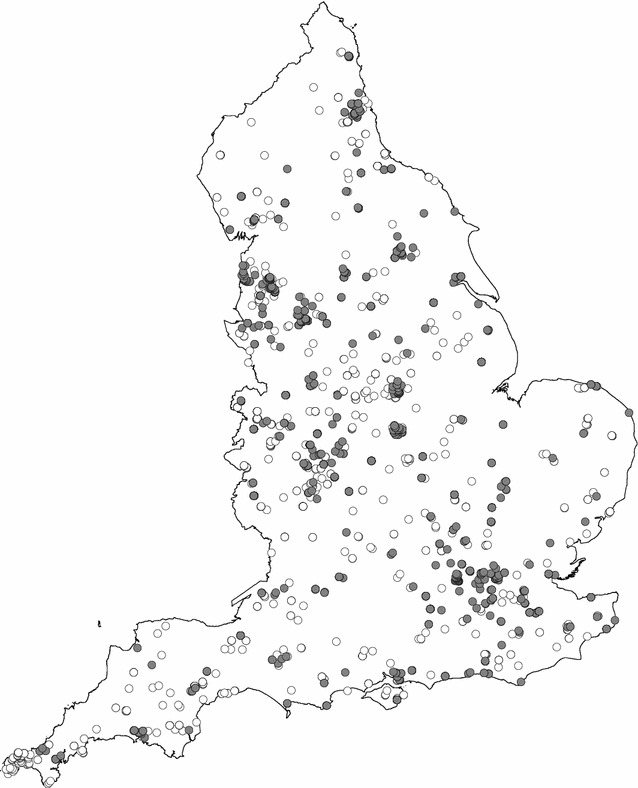
Map showing the distribution of completed OPAL Biodiversity Surveys across England between September 2010 and August 2012. *Solid grey circles* indicate surveys from urban areas; *open circles* indicate surveys from rural areas

Between September 2010 and August 2012, 2949 completed OPAL Biodiversity Surveys were returned, 82 % were recorded between April and October (83 % rural, 81 % urban). Figure 1 shows the distribution of survey returns across England. A total of 2891 responses that included both hedgerow and invertebrate data were included in the following analyses. Of these 46.6 % were on urban hedges with the remainder (53.4 %) on rural hedges. The majority of returns were from school groups (79.9 % urban, 60.5 % rural).

Recorded urban hedges were dominated by those in schools (55.3 %) and in gardens and parks (23.9 %), while rural hedges were dominated by those from farmland (30.8 %), schools (28.3 %) and grassland (22.3 %). Approximately one third of recorded hedges had no gaps (39.0 % urban, 34.2 % rural). In the entire sample, 69.5 % of hedges were recorded as untrimmed. In respect of other features of the boundary, 53.4 % also contained fences, 17.0 % ditches, 15.6 % earth banks, 27.2 % undisturbed strips and 11.7 % walls. 63.2 % of hedges were taller than 2 m and 43.8 % of hedges were wider than 2 m. Holes at the base of the hedge were reported in half of the returns (49.2 %).

### Woody species

Table 3 summarises the percentage of urban and rural hedges containing each recorded woody species and the mean number of woody species recorded at each site. All species, except Hazel, differed significantly between urban and rural sites. Beech, Holly, Ivy, Laurel, Privet and Yew were recorded more often in urban hedges, while Blackthorn, Bramble, Dog Rose, Elder and Hawthorn were recorded more often in rural hedges. There was no significant difference in the mean number of woody species recorded in urban and rural hedges.

**Table 3:** The percentage of urban and rural hedges containing the 12 recorded woody species

	Urban	Rural	χ^2^	P
Beech	23.1	13.9	38.13	<0.001
Blackthorn	25.9	31.7	10.90	0.001
Bramble	48.4	64.3	68.31	<0.001
Dog Rose	19.2	26.1	17.39	<0.001
Elder	15.6	19.3	6.35	0.012
Hawthorn	46.2	63.5	79.99	<0.001
Hazel	19.0	18.4	0.17	0.676
Holly	29.7	17.2	59.25	<0.001
Ivy	42.0	37.9	4.58	0.032
Laurel	17.1	6.1	82.56	<0.001
Privet	21.0	7.4	105.54	<0.001
Yew	8.9	4.1	26.73	<0.001
Mean number of woody species	3.16	3.10	0.92^a^	0.358

Significance is tested by Chi squared contingency tables, except mean number of species tested by independent samples *t* test

^a^ t statistic

### Invertebrates

Spiders were the most frequently recorded invertebrate, present in over half of all hedges surveyed. Table 4 summarises the percentage of urban and rural hedges that contained each invertebrate group, and the mean numbers of invertebrate groups and of the three calculated scores. Nine of the 24 invertebrate groups differed significantly between urban and rural hedges. Ants, earwigs and shieldbugs were recorded more often in urban hedges and blowflies, caterpillars, harvestmen, other beetles, spiders and weevils were recorded more often in rural hedges. There was no significant difference in the mean number of invertebrate groups recorded in urban and rural hedges.

**Table 4:** The percentage of urban and rural hedges containing the 24 recorded invertebrate groups

	Urban	Rural	χ^2^	P
Aphid	20.4	22.7	2.03	0.155
Ant	33.4	25.0	24.29	<0.001
Blowfly	9.2	13.1	10.33	0.001
Bee	13.4	13.3	0.01	0.912
Butterfly	10.4	10.1	0.08	0.779
Caterpillar	11.8	15.7	8.82	0.003
Centipede	6.7	5.6	1.52	0.218
Cranefly	7.1	8.6	2.14	0.143
Earwig	15.7	11.6	10.07	0.002
Froghopper	7.6	9.4	2.94	0.086
Harvestman	11.1	13.5	3.85	0.050
Hoverfly	9.8	9.9	0.01	0.936
Lacewing	4.8	4.6	0.08	0.773
Ladybird	23.2	23.4	0.02	0.889
Millipede	6.3	7.2	0.92	0.337
Moth	8.4	10.2	2.00	0.157
Other beetles	16.6	21.0	8.71	0.003
Shieldbug	9.1	6.3	7.83	0.005
Slug	12.5	11.6	0.47	0.492
Snail	25.2	25.9	0.18	0.675
Spider	54.5	58.8	5.17	0.023
Wasp	14.2	13.0	0.83	0.363
Weevil	6.3	9.5	9.72	0.002
Woodlouse	23.7	21.0	3.03	0.82
Mean number of invertebrate groups	3.61	3.71	−0.66^a^	0.513
Mean hedge structure score	23.1	24.4	−10.29^a^	<0.001
Mean wildlife food score	21.6	25.5	−7.16^a^	<0.001
Mean animal diversity score	17.6	19.1	−2.72^a^	0.007

Significance is tested by Chi squared contingency tables, except mean number of groups, and mean scores tested by independent samples *t* test

^a^ t statistic

The Spearman rank correlation between the number of woody species and number of invertebrate groups was 0.146 (p < 0.001) suggesting that more botanically diverse hedges were also more diverse in invertebrates.

### Hard surfaces

Only 5.3 % of the sample of 2891 hedges had hard surfaces on both sides. Hard surfaces were present on one or both sides for 45.2 % of urban hedges and 26.7 % of rural hedges.

**Fig. 2:**
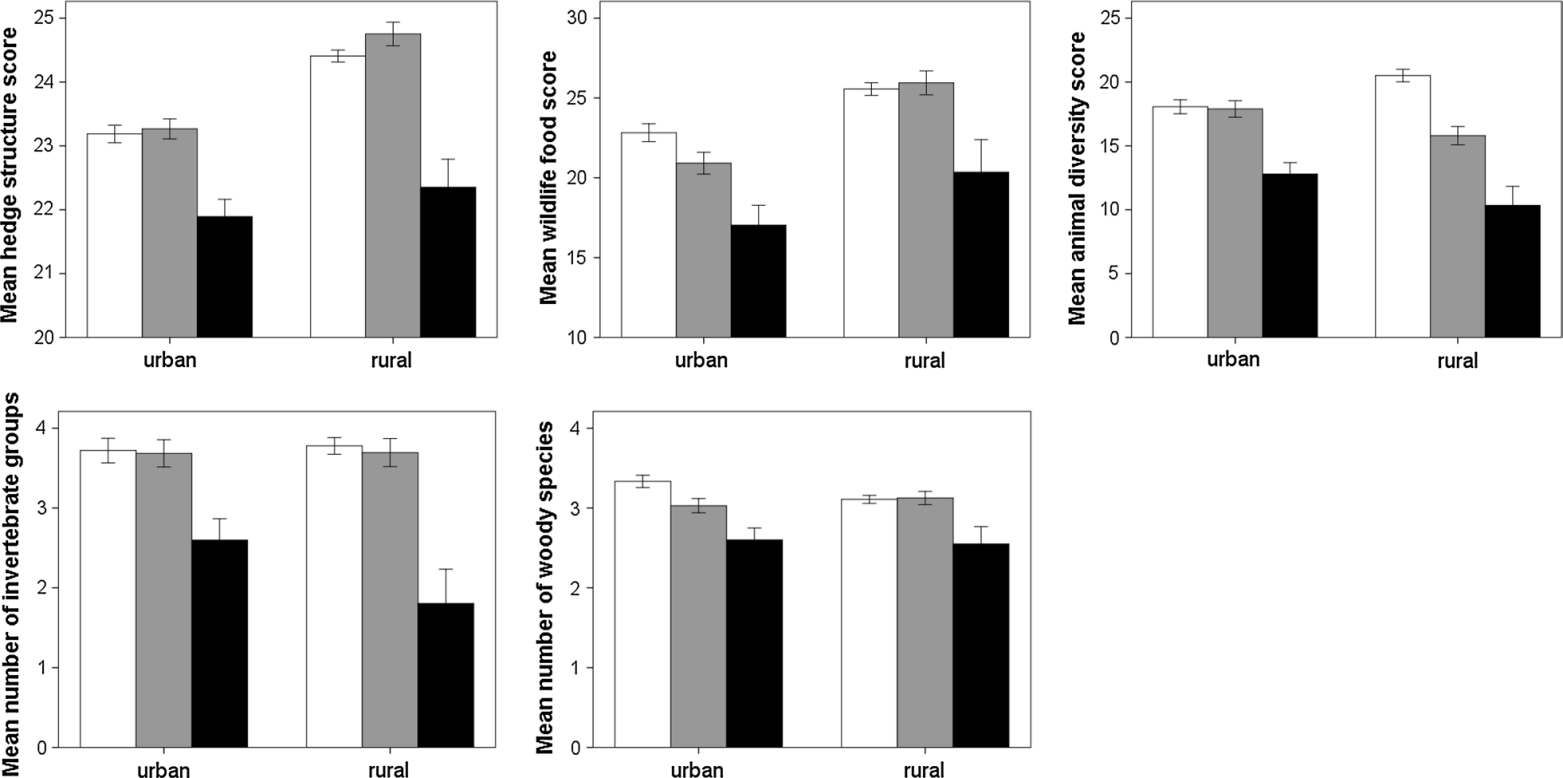
Mean ± SE scores displayed for hedge structure, wildlife food and animal diversity; and numbers of invertebrate groups and woody species for urban and rural hedges. *White bars* show data for hedges with no surrounding hard surfaces, *grey bars* for hedges with a hard surface on one side and *black bars* for hedges with a hard surface on both sides

Overall, rural hedges had a significantly higher structure score and wildlife food score than urban hedges (Table 5). Hedges with hard surfaces on both sides had significantly reduced scores for structure, wildlife food, and animal diversity, and much lower numbers of invertebrate groups and woody species (Table 5; Fig. 2). The interaction of hard surfaces and urban-rural was significant for two of these variables. For wildlife food score, mean values were lower in urban hedges with one hard surface compared to none, while in rural hedges there was little difference between these categories. However, for the animal diversity score, rural hedges with one hard surface had a lower mean than those with no hard surface, while in urban areas there was little difference between these two groups (Fig. 2).

**Table 5:** F statistics and p values from the ANOVAs to examine for urban-rural differences, the effects of adjacent hard surface, and their interaction on three scores and two measures of wildlife richness

		Hedge structure score		Wildlife food score		Animal diversity score		Number of invertebrate groups		Number of woody species	
	df_1_	F	P	F	P	F	P	F	P	F	P
Urban/Rural (UR)	1	21.33	<0.001	14.96	<0.001	0.47	0.491	0.97	0.324	0.25	0.618
Hard surfaces (H)	2	16.04	<0.001	8.57	<0.001	19.47	<0.001	10.06	<0.001	8.04	<0.001
UR*H	2	1.31	0.269	1.85	0.158	7.19	0.001	0.76	0.469	2.26	0.104

Numerator degrees of freedom as shown (df_1_), denominator d.f. ranged from 2691 to 2885

## Discussion

The results of this study have indicated that there are differences between urban and rural hedges and that adjacent hard surfaces may have an impact on hedge biodiversity.

The differences in woody species in urban and rural hedges (Table 3) are likely to reflect varying management practices. In urban areas, the likely preference for non-spiny shrubs in public areas such as parks and especially school grounds may determine the species commonly found. Not least, planted hedges found in urban areas are likely to contain shrubs that have dense foliage for privacy [3] and are easy to manage.

Bramble was less common in urban hedges; it tends to be more difficult to control and its sharp prickles are unpopular. Although only focusing on hedges in gardens in urban areas, Smith et al. showed that Privet was a dominant species [56]; in our study it was also recorded more often in urban hedges by survey participants. The higher wildlife food score found in rural hedges was clearly related to the plant species more commonly reported in rural areas, with four of the five species significantly more common in rural hedges scoring the highest possible value for wildlife food. Rural hedges also had a significantly higher structure score. The role that hedges perform—both now and historically—affects their structure [28]. Although the structure score was derived from a number of factors (Table 2), it could be assumed that rural hedges are longer, perhaps as field boundaries, and are more likely than urban hedges to be untrimmed, both of which generate higher scores.

The average number of woody species for urban hedges was 3.16. In contrast, Smith et al. found that 82 % of urban garden hedges contained only one plant species [56], although that figure covers hedges of varying lengths and some may have been shorter than 3 m.

The urban and rural differences in hedge plant composition highlighted by the survey have ecological implications, particularly for urban areas. The lower score for wildlife food in urban areas suggests that urban hedges provide fewer resources (flowers, seeds, fruits) for animals than their rural counterparts. A more heterogeneous hedge planting regime by residents and authorities may encourage more animals to make use of hedge habitats for food and shelter.

The difference between the presence of urban and rural invertebrates is more difficult to explain, partly because, despite the plethora of research on urbanisation and varying invertebrate assemblages (e.g. [57, 58]), the overall results are inconclusive. Urbanisation appears to have a positive or negative effect on invertebrates depending on the species. The OPAL Biodiversity Survey looked at invertebrates in broader taxonomic groups and therefore the results from this study are unlikely to provide insights into individual species preference for an urban or rural environment. In a review of research on the effect of urbanisation on flora and fauna, 29.8 % of studies that looked at the effects on invertebrates demonstrated an increase in species richness with increasing urbanisation, with 63.8 % showing a decrease and the remainder showing no change [59]. More investigation is needed and perhaps an alteration to the Biodiversity Survey methodology that would identify key indicator species before these results can provide any interpretable information regarding invertebrate preferences for urban or rural environments.

It is noted that when asking non-experts to identify invertebrates, a likely bias will occur towards those that are visually distinctive, as evidenced by Ward [60] for Hymenoptera in a New Zealand-based citizen science project. Spiders were the most frequently recorded invertebrate and this may be due to the relative ease of identifying them since they are distinctive from the remaining groups in the survey and more people are familiar with them. Other groups may also be more camouflaged than spiders, so participants may miss their presence or the invertebrates are able to flee the surveyor quickly before identification and recording can occur. It is also noted that seasonal differences and daily weather conditions are likely to have affected species presence and abundance.

The weak but highly significant positive correlation between increasing numbers of woody species and an increase in invertebrate diversity is supported by a large volume of existing research (see [24], and references supplied therein for a summary).

Results from the OPAL Biodiversity Survey suggest that the presence of hard surfaces on both sides of the hedge can have a significant impact on the biodiversity it supports. Furthermore, results have shown that even just one side of the hedge being a hard surface can have some impact. This is an important result as it suggests that many of the hedges that we see alongside roads and in much of the urban landscape may not have as much wildlife value as the presence of the hedge itself alone would imply.

Existing research identifies the environmental impacts of hard surfaces, often focusing on the general loss of green space in urban areas (e.g. [61]), but few specifically discuss the impact of hard surfaces on hedge biodiversity. However, Faiers and Bailey [33] noted, in their study on canalside hedges, that surrounding amenity value (the potential of the site to accommodate visitors e.g. footpaths, car parks) were negatively correlated with the biodiversity and structure value of the hedge. Although it is not possible to ascertain the number of adjacent hard surfaces to the study hedges, the investigation by Faiers and Bailey does support the finding that a hard surface—whatever its use—may have a negative impact on hedges. Smith et al. [62], in their study of urban garden habitats, also found that the presence of hard surfaces was negatively correlated with the abundance of some invertebrates.

Other studies have shown that surrounding habitat types in general impact upon the invertebrate diversity within hedges. Dover and Sparks [16] demonstrated that butterflies were more abundant in hedges adjacent to woodland and areas where floral density was high. Croxton et al. [63] found that the inside of green lanes (tracks with hedges on both sides) had higher bumblebee abundance and richness, suggesting that “two hedges are better than one”. In addition, that study also demonstrated that plant assemblages differed, with the inside of tracks a better resource for wildlife. Although these studies did not specifically concern hard surfaces adjacent to the hedge, they demonstrated that habitat types surrounding hedges can have a significant impact on the diversity found within.

The topic of the impact of adjacent hard surfaces on hedges is one that may need further research, particularly for hedges in urban areas.

There are a number of factors that can affect the quality of data resulting from a large citizen science survey such as the OPAL Biodiversity Survey. While it is beyond the scope of the present paper to discuss these factors in detail they should nevertheless be acknowledged. The majority of surveys are carried out by untrained individuals in their own time and in their local area, therefore it is impossible for the results of the survey to be verified by experts. Bonney [64] states that ensuring participants have clear instructions, guidance and data forms are important for accurate data submission. Rigorous testing of the survey was carried out and shaped the final draft while provision of face-to-face training also helped to improve the quality of data submitted. These aspects do not guarantee data accuracy; however they are key elements to the OPAL Biodiversity Survey. Other studies involving OPAL surveys have been undertaken to test the validity of results from citizen scientists. Rose et al. [65] discussed these at length and described the verification tests used for data submitted for the OPAL Water Survey. They noted that when testing variability in sampled water invertebrate results from different experienced surveyors, there was a reasonably high level of variability, although this reduced when results were amalgamated for the whole pond. Furthermore, when comparing results from untrained participants with those from experts, they found that they matched reasonably well. A comparison between OPAL Soil and Earthworm Survey results and those obtained from national databases showed that there was a reasonable match [66]. Similarly, a study to assess the usefulness of results from the OPAL Air Survey demonstrated that the methodology employed by the survey could indicate presence of nitrogenous air pollution but not at low concentrations [67].

Although covering different environmental topics, these studies suggest that while the survey methods employed by OPAL may not be suitable for measuring small-scale phenomena, when applied on a broad scale, they are of value. Furthermore, when coupled with the educative aims of the surveys, they offer considerable value.

## Conclusions

This study has shown that urban and rural hedges are different in floral and faunal composition and that adjacent hard surfaces may have an impact on hedge biodiversity. The constraints of the survey methodology and associated data do not allow for detailed investigation, however, the findings have implications on how urban hedges in particular are managed, with suggestion that the surfaces immediately adjacent to the hedge need consideration if the wildlife value of an urban hedge is to be optimised. Overall, the study has highlighted the need for more research to be undertaken on the under-recorded topic of urban hedges and the effects that adjacent hard surfaces may have on their biodiversity. In addition, the OPAL Biodiversity Survey, as with other OPAL surveys, has shown that the public have enthusiasm for completing simple ecological surveys. Despite the limitations regarding data verification, the survey provides a basis for further research using citizen science methodology.

Utilising the manpower of the general public enables scientists some additional capacity to study hedge habitats. Furthermore, educating the public about hedges and the plants and animals that use them can help to protect their future. Encouraging people to develop a passion for the natural world and recording, monitoring and protecting it is perhaps one of the best future-proofing techniques against further habitat loss that scientists can provide.
